# Effect of alcohol on Brain-Derived Neurotrophic Factor (BDNF) blood levels: a systematic review and meta-analysis

**DOI:** 10.1038/s41598-023-44798-w

**Published:** 2023-10-16

**Authors:** Arman Shafiee, Kyana Jafarabady, Mohammad Ali Rafiei, Maryam Beiky, Niloofar Seighali, Golshid Golpayegani, Mehrsa Jalali, Faeze Soltani Abhari, Razman Arabzadeh Bahri, Omid Safari, Mahmood Bakhtiyari, Amirhesam Alirezaei

**Affiliations:** 1 Student Research Committee, School of Medicine, Alborz University of Medical Sciences, Karaj, Iran; 2 Department of Psychiatry and Mental Health, Alborz University of Medical Sciences, Karaj, Iran; 3 Student Research Committee, School of Medicine, Shahid Beheshti University of Medical Sciences, Tehran, Iran; 4 School of Medicine, Tehran University of Medical Sciences, Tehran, Iran; 5 Non-Communicable Diseases Research Center, Alborz University of Medical Sciences, Karaj, Iran; 6 Department of Community Medicine and Epidemiology, Alborz University of Medical Sciences, Karaj, Iran; 7 Department of Nephrology, Shahid Beheshti University of Medical Sciences, Tehran, Iran

**Keywords:** Neuroscience, Psychiatric disorders

## Abstract

Brain-Derived Neurotrophic Factor (BDNF) is a vital protein involved in neuronal development, survival, and plasticity. Alcohol consumption has been implicated in various neurocognitive deficits and neurodegenerative disorders. However, the impact of alcohol on BDNF blood levels remains unclear. This systematic review and meta-analysis aimed to investigate the effect of alcohol consumption on BDNF blood levels. A comprehensive search of electronic databases was conducted to identify relevant studies. Eligible studies were selected based on predefined inclusion criteria. Data extraction was performed, and methodological quality was assessed using appropriate tools. A meta-analysis was conducted to estimate the overall effect size of alcohol consumption on BDNF levels. A total of 25 studies met the inclusion criteria and were included in the final analysis. Alcohol use and BDNF blood levels were significantly correlated, according to the meta-analysis (*p* = 0.008). Overall, it was discovered that drinking alcohol significantly decreased BDNF levels (SMD: − 0.39; 95% CI: − 0.68 to − 0.10; I2: 93%). There was a non-significant trend suggesting that alcohol withdrawal might increase BDNF levels, with an SMD of 0.26 (95% CI: − 0.09 to 0.62; I2: 86%; *p* = 0.14). Subgroup analysis based on the source of BDNF demonstrated significant differences between the subgroups (*p* = 0.0008). No significant publication bias was observed. This study showed that alcohol consumption is associated with a significant decrease in BDNF blood levels. The findings suggest a negative impact of alcohol on BDNF levels regardless of alcohol dosage. Further studies are needed to strengthen the evidence and elucidate the underlying mechanisms.

## Introduction

Brain-Derived Neurotrophic Factor (BDNF) is a crucial protein that plays a vital role in the development, survival, and plasticity of neurons in the central nervous system^1^. It is involved in various neurobiological processes, including neuronal growth, differentiation, and synaptic plasticity^2^. BDNF is widely expressed in the brain, and its dysregulation has been implicated in the pathophysiology of numerous neurological and psychiatric disorders, such as depression, Alzheimer's disease, and substance use disorders^2,3^.

Alcohol consumption is a prevalent and culturally accepted practice in numerous societies^4^. It has been extensively studied for its detrimental effects on various organ systems, including the brain^5^. Chronic and excessive alcohol consumption is associated with a range of neurocognitive deficits, neurodegeneration, and increased vulnerability to psychiatric disorders^6^.Recent research has focused on elucidating the molecular mechanisms underlying alcohol's impact on brain function, with an increasing interest in its effects on BDNF^7^.

The association between alcohol consumption and BDNF levels has gained substantial attention due to its potential implications for understanding the neurobiological effects of alcohol on brain health^8^. Preclinical studies have demonstrated that alcohol exposure can modulate BDNF expression, leading to alterations in neuronal plasticity and impairments in cognitive functions^8,9^. Additionally, clinical studies have provided evidence of BDNF dysregulation in individuals with alcohol use disorders, further highlighting the importance of investigating the relationship between alcohol and BDNF levels^10^.

However, the existing literature on the effect of alcohol on BDNF blood levels is characterized by inconsistent findings, which may be attributed to variations in study design, participant characteristics, alcohol consumption patterns, and BDNF measurement methods. Therefore, this study aims to synthesize the current literature on the effect of alcohol consumption on BDNF blood levels through a rigorous and comprehensive analysis.

## Method

### Study protocol and search strategy

A comprehensive research plan was formulated to provide guidance throughout the systematic review procedure and was registered in PROSPERO under the registration number CRD42023433709. This systematic review followed the guidelines outlined in the Preferred Reporting Items for Systematic Reviews and Meta-Analyses (PRISMA) to ensure transparent and comprehensive reporting^11^.

### Search strategy

Electronic databases including PubMed, Embase, Scopus, and PsycINFO were searched from inception until 5 June 2023. The search terms used were "alcohol," "ethanol," "BDNF," "Brain-Derived Neurotrophic Factor," and relevant synonyms and variations. No language restrictions were applied (Supplementary file). Additionally, the reference lists of relevant articles and review papers were manually searched to identify additional studies.

### Study selection

Two independent reviewers screened the titles and abstracts of the retrieved articles to assess their eligibility for inclusion. Full-text articles were retrieved for potentially relevant studies or when the title and abstract provided insufficient information. Studies were considered for inclusion if they met the following criteria: (1) examined the effect of alcohol consumption on BDNF blood levels; (2) included human participants; and (3) presented original data. Studies that did not meet these criteria or were review articles, case reports, or animal studies were excluded. Any disagreements between the reviewers were resolved through discussion and consensus.

### Data extraction

Data extraction was conducted independently by two reviewers using a standardized data extraction form. The following information was extracted from each included study: author(s), year of publication, study design, sample size, participant characteristics (e.g., age, sex), alcohol consumption patterns (e.g., definition or time), BDNF measurement methods, and BDNF blood level data (e.g., mean, standard deviation, or other effect size measures). Any discrepancies in data extraction were resolved through discussion and consensus.

### Quality assessment

The methodological quality and risk of bias of the included studies were assessed using appropriate tools. The Newcastle–Ottawa Scale (NOS) for cohort and case–control^12^. NOS is mostly used to evaluate the quality of nonrandomized studies such as cohort and case–control studies^13^. NOS includes four domains as followed: selection of participants domain, comparability domain, the ascertainment of exposure (for case control studies) or outcome of interest (for cohort studies). Selection of exposed and nonexposed participants, exposure ascertainment and presence of outcome of interest at the beginning of study are determined in selection and ascertainment domain. In comparability number of cofounders that were adjusted in evaluated. Appropriateness of methodology that evaluate the outcomes, rate of follow up loss and length of follow up are determined in outcome of interest domain^13^. Star rating system is used in NOS, maximum nine stars can be given to a study. Studies with 7–9 scores are rated as “Good quality”, 4–6 scores as “Fair quality”, and lower than 3 are rated as “Poor quality”. Each study was independently assessed by two reviewers, and any disagreements were resolved through discussion and consensus.

### Data synthesis and analysis

A meta-analysis was conducted to estimate the overall effect size of alcohol consumption on BDNF blood levels. The standardized mean difference (SMD) was used as the effect size metric due to the variability in BDNF measurement methods across studies. A random effect model was used to account for potential heterogeneity among the included studies. Heterogeneity was assessed using the I^2 statistic, where values of 25%, 50%, and 75% represent low, moderate, and high heterogeneity, respectively. Subgroup analyses were performed based on the source of BDNF and duration of withdrawal. Publication bias was assessed visually using funnel plot asymmetry and statistically using Egger's regression test. Funnel plot asymmetry may indicate the presence of publication bias, with a symmetric plot suggesting the absence of bias. Egger's regression test assesses the relationship between effect size and its precision, with *p* < 0.05 considered indicative of significant publication bias. All analysis were conducted in StataCorp. 2015. Stata Statistical Software: Release 14. College Station, TX: StataCorp LP and RevMan 5.3.

## Results

**Figure 1:**
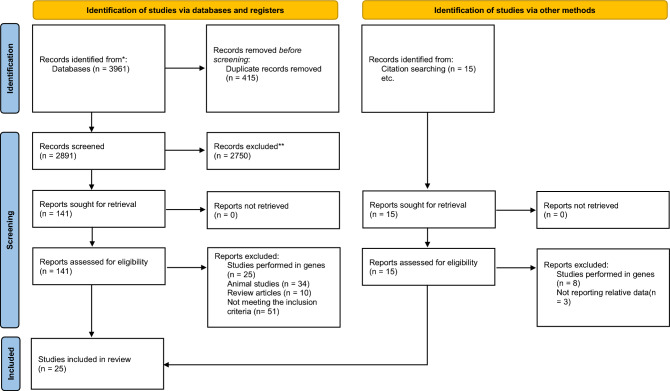
PRISMA flow diagram.

The present systematic review and meta-analysis aimed to investigate the effect of alcohol consumption on Brain-Derived Neurotrophic Factor (BDNF) blood levels. A comprehensive search of electronic databases was conducted, and a total of 25 studies met the inclusion criteria and were included in the final analysis ^14–38^ (Fig. 1). The studies were published between 2007 and 2021. There were 10 studies that assessed the effect of alcohol withdrawal programs on BDNF levels^29–38^ (Table 1). All studies were rated good or fair regarding their quality (Supplementary Table 3).

**Table 1:** Characteristics of included studies.

Author, year	Country	Study design	BDNF measurement tool	Source (serum, plasma)	Number of participants	Definition for alcohol use disorder	Time of being alcoholic	Age (mean + SD)	Male (%)	Withdrawal	Quality
A. C. Lhullier (2015)	Brazil	Cross-sectional	DuoSet ELISA Development, R&D systems, minneapolis, MN	Serum	Cases = 77 Controls = 718 Total = 795	CAGE questionnaire	–	26.06 5.14	0.4352	–	GOOD
Bus, B. A. (2011)	the Netherlands	Cohort study	NR	Serum	Cases = 1168	NR	NR	18–65	NR	No	Good
Cavus, S. Y. (2012)	Turkey	Case control	Sandwich enzyme-linked immunosorbent essay (ELISA)	Serum	Cases = 31 Controls = 29 Total = 60	DSM-IV-TR	NR	18–65	Cases = 100% Controls = 100%	Yes	Good
Costa, M. A. (2011)	France	Case control	NR	Serum	Cases = 101 Controls = 41 Total = 142	Clinical signs and measured levels of biological markers of alcohol consumption	NR	NR	NR	Yes	Poor
Garcia-Marchena, N. (2017)	Spain	Case control	Bio-Plex Suspension Array System 200 (Bio-Rad Laboratories, Hercles, CA, USA)	Plasma	Cases = 91 Controls = 55 Total = 146	DSM-IV-TR	13 years	Cases = 47.91 (7.69) Controls = 45.15 (10.06)	Cases = 67% Controls = 65.5%	No	Good
Geisel, O. (2016)	Germany	Case control	NR	Serum	NR	NR	NR	18–65	NR	Yes	Fair
Gorka, S. M. (2020)	USA	Case control	Enzyme-linked immunosorbent assay kit (BioVision, Milpitas, CA)	Plasma	Cases = 34 Controls = 23 Total = 57	DSM-5 Disorders	NR	Cases = 23.6 Controls = 24.0	Cases = 60.6% Controls = 45.8% Total = 54.4%	No	Good
Heberlein, A. (2010)	Germany	Case control	CIWA-Ar score	Serum	Cases = 81 Controls = 41 Total = 122	NR	NR	NR	Cases = 100% Controls = 100%	Yes	Poor
Heberlein, A. (2014)	Germany	Case control	NR	Serum	Cases = 30 Controls = 18 Total = 48	NR	NR	NR	Cases = 100% Controls = 100%	Yes	Poor
Heberlein, A. (2016)	Germany	Case control	NR	Serum	Cases = 99 Controls = 17 Total = 116	NR	NR	NR	Cases = 100% Controls = 100%	Yes	Poor
Huang, M. C. (2008)	Taiwan	Case control	Sandwich enzyme-linked immunosorbent essay	Serum	Cases = 25 Controls = 22 Total = 47	DSM-IV	8.5 ± 5.8 years	Cases = 41.3 ± 7.8 Controls = 43.6 ± 6.3	Cases = 84% Controls = 86.3%	Yes	Good
Huang, M. C. (2011)	Taiwan	Case control	Sandwich enzyme-linked immunosorbent essay	Serum	Cases = 65 Controls = 39 Total = 104	DSM-IV	NR	NR	NR	Yes	Fair
Joe, K. H. (2007)	Korea	Case control	Duo- Set ELISA Development System (DY248; R&D Systems, U.K.)	Plasma	Cases = 64 Controls = 75 Total = 139	Diagnostic and Statistical Manual IV (DSM-IV)	332.4 ± 120.5 months	Cases = 47.62 ± 10.56 Controls = 34.3 ± 11.5	Cases = 100% Controls = 100%	30 Days	GOOD
Kethawath, S. M. (2020)	India	Case control	The RayBio Human BDNF ELISA kit, Ray Biotech Ltd., Norcross, Georgia, USA	Serum	Cases = 25 Controls = 25 Total = 50	ICD-10 criteria	8.8 (± 7.8)	Cases = 38.2 (± 8.5) Controls = 36.0 (± 11.6)	Cases = 100% Controls = 100%	1 to 10 Days	GOOD
Kohler, S. (2013)	Germany	Case control	ELISA kit (Promega Inc., Mannheim, Germany)	Plasma	Cases = 15 Controls = 15 Total = 30	Diagnosis of alcoholism according to DSM IV criteria	17.6 ± 9 years	Cases = 48.8 ± 7 Controls = 48.6 ± 7	Cases = 73.3% Controls = 73.3%	1 to 14 Days	GOOD
Lee, B. C. (2009)	Hangang	Case control	DuoSet enzyme-linked immunosorbent assay (ELISA) (Catalog number DY248 and DY256, R&D Systems, UK)	Plasma	Cases = 41 Controls = 41 Total = 82	Diagnostic and Statistical Manual of Mental Disorder, Fourth Edition (DSM-IV) criteria	12.1 6 10.4	Cases = 48.3 ± 10.1 Controls = 45.2 ± 9.2	Cases = 100% Controls = 100%	24 HOURS	GOOD
Martin-Gonzalez, C. (2021)	Spain	Case control	Quantitative sandwich enzyme immunoassay technique	Serum	Cases = 82 Controls = 27 Total = 109	–	33 ± 14	Cases = 58.62 ± 11.21 Controls = 54.52 ± 7.78	Cases = 100% Controls = 100%	–	GOOD
Meng, D. (2011)	Southwestern	Case control	Sandwich ELISA kit (ChemiKine, Millipore Corp.)	Serum	Cases = 14 Controls = 10 Total = 24	Diagnostic and Statistical Manual of Mental Disorders– Version IV (DSM-IV) criteria	18.9 ± 8.7	Cases = 39.6 ± 10.3 Controls = 37.5 ± 11.9	Cases = 100% Controls = 100%	4—6 weeks	GOOD
Silva-Peña, D. (2019)	Spain	Cross-sectional	Enzyme-linked immunosorbent assay (ELISA) (#ab212166, Abcam, Cambridge, UK)	Plasma	Cases = 58 Controls = 22 Total = 80	Diagnostic and Statistical Manual of Mental Disorders—4Version IV (DSM-IV- TR) criteria	_	Cases = 49.22 (8.20) Controls = 47.27 (10.73)	Cases = 82.8% Controls = 86.4%	≥ 4 weeks of abstinence	GOOD
Sonmez, M. B. (2016)	Turkey	Case control	Enzyme-linked immunosorbent assay (ELISA) kits (Boster Biological Tech- nologies, CA, USA)	Serum	Cases = 34 Controls = 32 Total = 66	Statistical Manual of Mental Disorders, Fourth Edition (DSM-IV-TR)	16.32 ± 8.82	Cases = 45.44 ± 8.98 Controls = 41.78 ± 13.15	Cases = 100% Controls = 100%	–	GOOD
Wilhelm, C. J. (2017)	Portland	Case control	Myriad rules based medicine, Inc., a CLIA certified laboratory, using a human inflammation multi-analyte Profile (v 1.0) panel designed to discern inflammatory patterns in biological samples (Austin, TX, USA)	Plasma	Cases = 51 Controls = 31 Total = 82	Diagnostic and Statistical Manual of Mental Disorders– Version IV (DSM-IV) criteria (American Psychiatric Association, 2000)	Male : average use > 6 standard drinks/day for ≥ 1 year Female: average use > 4 standard drinks/day for ≥ 1 year	Male : Cases = 36.2 (10.6) Controls = 41.3 (13.9) Female : Cases = 34.7 (10.6) Controls = 29.7 (10.3)	Cases = 78.43% Controls = 67.74%	–	GOOD
Xu, Y. Y. (2020)	China	Case control	Commercially available enzyme-linked immunosorbent assay (ELISA) kits	Plasma	Cases = 83 Controls = 61 Total = 144	International Classification of Diseases 10th Revision (ICD-10) criteria	7.56 ± 6.62	Cases = 39.16 ± 9.06 Controls = 42.39 ± 11.41	Cases = 100% Controls = 100%	1 DAY	GOOD
Zanardini, R. (2011)	Italy	Case control	ELISA with the human BDNF Quantikine kit (cat. DBD00; R&D Systems Minneapolis)	Serum Plasma	Cases = 37 Controls = 37 Total = 74	Diagnostic and Statis- tical Manual of Mental Disorders-IV (DSM-IV) criteria	7.65 years	Cases = 50.11 ± 11.05 Controls = 50.00 ± 10.93	Cases = 67.56% Controls = 67.56%	–	GOOD
Zhang, X. Y. (2016)	China	Case control	Sandwich ELISA	Serum	Drinking only (case1) = 31 Non-smoking + non-drinking (control1) = 55 Smoking + drinking (case2) = 58 Smoking only (control2) = 47	Axis I disorders on Diagnostic and Statistical Manual of Mental Disorders—Fourth Edition (DSM-IV)	–	Drinking only (case1) = 43.4 ± 16.5 Non-smoking + non-drinking (control1) = 41.6 ± 17.0 Smoking + drinking (case2) = 42.2 ± 13.3 Smoking only (control2) = 45.0 ± 14.7	Cases = 100% Controls = 100%	–	GOOD
Zhou, L. (2017)	China	Case control	ELISA		Cases = 30 Controls = 50 Total = 80	International Statistical Classification of Diseases and Related Health Problems 10th Revision (ICD-10) criteria	16.971 ± 5.8	Cases = 43.891 ± 7.522 Controls = 43.696 ± 8.773			GOOD

### Effect of alcohol on BDNF blood levels

**Figure 2:**
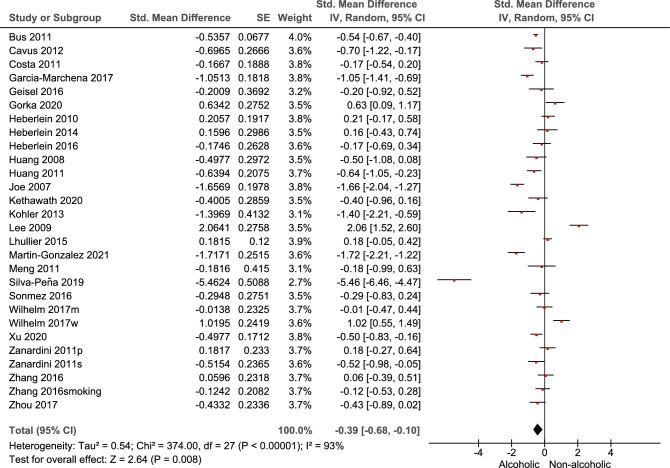
Results of meta-analysis for the level of Brain-Derived Neurotrophic Factor (BDNF) levels in alcohol use disorder.

**Figure 3:**
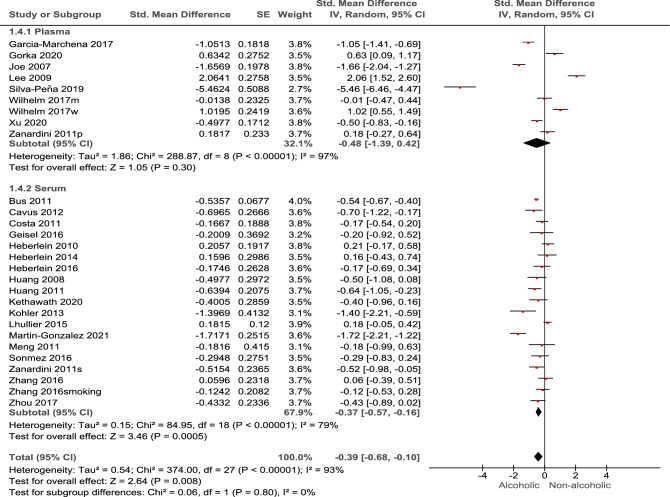
Results of subgroup analysis.

The findings from the meta-analysis revealed a significant association between alcohol consumption and BDNF blood levels (*p* = 0.008). Overall, alcohol was found to have a significant negative effect on BDNF levels, (SMD: − 0.39; 95% CI: − 0.68 to − 0.10; I2: 93%). This indicates that alcohol consumption is associated with a decrease in BDNF blood levels (Fig. 2). Subgroup analysis based on the source of BDNF showed only the serum BDNF was significantly reduced when comparing alcoholic patients and healthy controls (SMD: − 0.37; 95% CI: − 0.57 to − 0.16; *p* = 0.008), however it should be noted there was a non-significant between-subgroup differences (*p* = 0.80) (Fig. 3). The results of meta regression investigating the possible correlation between age and % of male participants on the overall effect size only showed a significant correlation with regard to age with a negative correlation (Coefficient: − 0.081) (Table 2).

**Table 2:** Results of meta-regression for comparing the level of BDNF between alcoholic patients and healthy controls.

	Coefficient	Standard error	P >|z|	95% CI
Meta-regression				
Age	− 0.081	0.025	**0.002**	− 0.13 to − 0.03
Male (%)	0.0019	0.006	0.781	− 0.011 to 0.016

Signifiant value in bold.

### Effect of alcohol withdrawal program on BDNF blood levels

**Figure 4:**
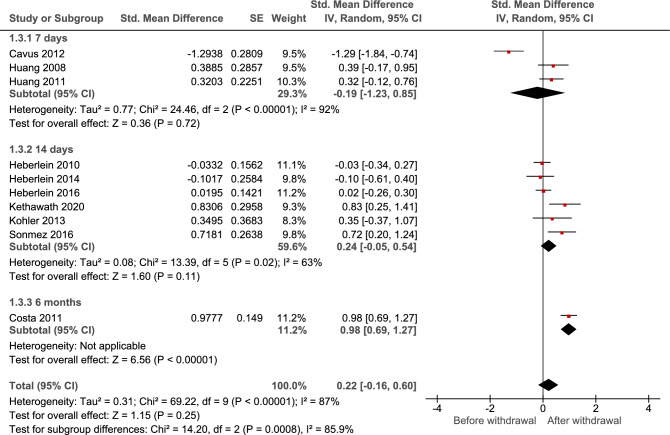
Results of meta-analysis for the level of Brain-Derived Neurotrophic Factor (BDNF) levels in alcohol withdrawal.

Alcohol withdrawal was found to have a non-significant trend for increasing the BDNF levels, (SMD: 0.26; 95% CI: − 0.09 to 0.62; I2: 86%; *p* = 0.14). Subgroup analysis based on the source of BDNF showed significant between-subgroup differences (*p* = 0.0008), showing significant increase in SMD among 6-month withdrawal group (SMD: 0.98; 95% CI: 0.69 to 1.27; *p* < 0.001) (Fig. 4). Future studies are warranted to investigate the effect of alcohol withdrawal on BDNF levels.

### Publication bias

**Figure 5:**
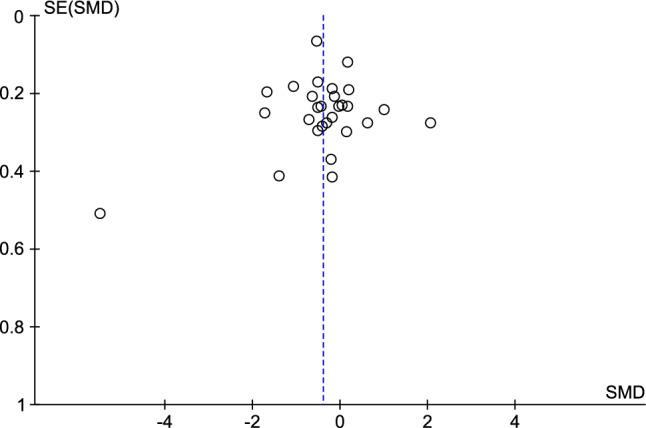
Funnel plot.

Publication bias was assessed using funnel plot visualization and Egger's regression test. The funnel plot appeared symmetrical, indicating no substantial evidence of publication bias (Fig. 5). Egger's regression test revealed no statistically significant publication bias (*p* = 0. 99).

## Discussion

In this study, we found a significant association between alcohol consumption and lower BDNF blood levels. Overall, the results of 25 included studies in this meta-analysis revealed that alcohol consumption is associated with a significant decrease in BDNF blood levels. In subgroup analysis based on the source of sampling, we found a significant association between alcohol consumption and lowered serum BDNF levels, while no significant association was observed between plasma BDNF levels and alcohol consumption. Moreover, the aforesaid subgroup analysis did not indicate significant between subgroup differences. While the impact of alcohol withdrawal on BDNF blood levels was not statistically significant overall, our subgroup analysis revealed that the effect of alcohol withdrawal on BDNF levels is supported by a higher level of evidence as the periods of withdrawal become longer. Furthermore, the between subgroup differences in subgroup analysis based on the length of withdrawal periods were significant.

### Measurement of BDNF levels in serum versus plasma

The accuracy of measuring BDNF levels in both plasma and serum can be subject to several factors, such as the type of anticoagulant used, duration of clotting, temperature, hormonal fluctuations, and the time elapsed before blood sample centrifugation^39–42^. It has been shown that average levels of BDNF in serum are higher than plasma BDNF levels^42^. This difference can be attributed to the amount of BDNF released from the platelets to the serum^43^. Furthermore, it is worth noting that while plasma BDNF levels may indicate the concentration of free circulating BDNF, serum BDNF levels are more closely linked to the overall BDNF amount contained in platelets and released in vitro during clotting. Therefore, serum BDNF levels may provide a more accurate reflection of the BDNF levels in whole blood^44^. Moreover, BDNF serum level seems superior in terms of stability^45–47^. In the context of our subgroup analysis based on the source of sampling, this information may be beneficial to explain the non-significant link between alcohol consumption and plasma BDNF levels. However, we should also consider other possible explanations for these results. For instance, the lower number of studies reporting plasma BDNF levels compared to serum BDNF levels may have contributed to these findings.

### Variables affecting BDNF peripheral levels

At the current state of the art, all origins of BDNF in human plasma are not yet fully recognized, but research suggests that they may also be produced in inflamed tissues^48^. This is particularly relevant for alcohol-dependent patients, who may have inflammation in various organs such as pancreas or liver^49–51^. Therefore, BDNF blood levels may reflect not only their levels in the brain but also the overall inflammatory state of the patient. This may affect the results of included studies and consequently our meta-analysis.

BDNF expression and its peripheral levels are affected by gender. In a study conducted by Lommatzsch et al., it was discovered that women had notably lower levels of BDNF in both their platelets and plasma compared to men^52^. However, after adjusting for body weight or BMI, there were no significant variations in plasma BDNF levels between the two genders^42^. Moreover, Piancatelli et al. found that a particular BDNF gene polymorphism, which may lead to a decrease in BDNF serum levels, is more prevalent among individuals with Alzheimer's disease. Interestingly, this association was particularly pronounced among female participants^53^. Furthermore, evidence suggests that steroid hormones and menstrual periods may have a regulatory effect on both expression and function of BDNF^40,54,55^. Accordingly, it is important to note that the differing ratios of male and female subjects in the included studies in this meta-analysis may contribute to heterogeneity and these concerns should be taken into account in future research.

Evidence suggests that BDNF levels are lower in individuals with depression, and antidepressant medications can increase BDNF levels^56–58^. Depression is characterized by hyperactive hypothalamo-pituitary-adrenocortical (HPA) axis, also BDNF is known as an essential mediator in regulation of HPA axis^59^. Interestingly, a neuroactive steroid named as allopregnanolone is also associated with anti-depressive effects, through regulation of HPA and also modulating BDNF central level^59,60^. In addition, neuroactive steroids, including allopregnanolone, can function in a sexual manner^61^. Therefore, it is crucial to consider the potential sex-specific interaction between neuroactive steroids and BDNF in order to gain a deeper understanding of the mechanisms underlying sex-related differences in BDNF levels.

### Unlocking alcohol dependence: the possible role of alternations in BDNF levels

Based on animal studies, chronic alcohol exposure may lead to a decrease in BDNF expression in certain brain structures, while repeated episodic exposure can result in an increase in BDNF expression. Additionally, repeated episodic exposures are associated with experiencing more withdrawal episodes^62,63^. Interestingly, Huang et al.'s investigation revealed that patients who experience delirium tremens (DT) after alcohol intoxication exhibit lower BDNF levels compared to those who do not develop DT. Thus, it is suggested that the rise of BDNF during withdrawal may be seen as a protective mechanism in response to alcohol withdrawal. However, aforementioned study found a significant increase in BDNF blood levels of subjects after one week of withdrawal in contrast to our results^64^. Moreover, some other investigators also found a significant increase in BDNF blood after withdrawal in alcohol consumers^65,66^. These may suggest that BDNF plays a role in neuronal remodeling after withdrawal and even maintenance of abstinence. Interestingly, McGough et al. found that an increase in BDNF blood level by RACK1 protein weakens behavioral effects of alcohol such as its consumption. Moreover, acute exposure to alcohol causes an increase in BDNF level. Thus, they hypothesized that BDNF is a part of regulatory system that antagonizes tolerance and subsequently addiction to alcohol^67^. More noticeable discoveries concerning the correlation between alcohol dependence and BDNF levels have been made through animal studies. One such study conducted by Jeanblanc et al. suggests that the activation of the MAPK pathway by BDNF may deter alcohol dependence^68^. Additionally, another investigation by the same researcher found that administering BDNF to the dorsolateral striatum (DLS) resulted in a decrease in voluntary alcohol consumption, indicating that BDNF plays a role in regulating alcohol intake^69^. In addition to this, we should note that patients with a positive family history of alcohol dependence have a lower mean BDNF blood levels that may demonstrate the probable role of BDNF in the pathophysiology of alcohol dependence^70^. Conduction of further studies investigating probable links between genes related to BDNF expression and alcohol dependence would be elucidative in this regard.

Ornell et al. conducted a systematic review of articles to evaluate BDNF blood levels in individuals with substance use disorders, including 13 articles that specifically examined BDNF levels in alcoholics. While our study included 25 articles between 2007 and 2021. Our findings regarding BDNF serum and plasma levels in alcohol users are consistent with Ornell et al.'s study, as they also reported a significant decrease in BDNF serum levels and a non-significant change in plasma BDNF levels among alcohol users. However, they observed a significant lower BDNF plasma level in alcohol users during withdrawal in contrast to our findings, which can be attributed to the fact that their analysis was based on a more limited number of included studies. Regarding the BDNF serum levels in withdrawal, their study did not reveal any significant finding^71^.

### Strength and limitations

Our review is strengthened by the development of a comprehensive systematic search protocol for obtaining up-to-date results from 4 databases, increasing the accuracy and reliability of our conclusions. Additionally, we obtained supplementary data from the studies to maximize the amount of analyzed data, and we analyzed and assessed the confidence level of each reported outcome. In order to analyze each outcome, we obtained information from at least 2 studies. In addition to finding a significant relationship between BDNF blood levels and alcohol consumption, we conducted several subgroup analyses regarding BDNF blood levels in alcohol users which yielded noteworthy findings ascertaining previous findings related to the probable link of BDNF to alcohol dependence pathophysiology.

However, our study faced limitations given the limited number of included studies especially in subgroup analysis, which may lead to bias and uncertainty of our results. Other limitations include differences in the duration of alcohol use and severity of dependance of subjects among included studies. Thus, several aspects of heterogenicity are noted in our study. It is critical to keep in mind that when evaluating a factor such as BDNF that has different levels in males and females, the resulting outcomes may differ between genders, potentially influencing the reliability of our findings. Moreover, our analysis is mainly based on cross-sectional studies which are limited in their ability to establish cause-and-effect relationships between alcohol use and BDNF peripheral levels. Additionally, these studies are unable to track changes in BDNF alternations over time, which limits their ability and as a consequence our meta-analysis to understand how these two may interact and evolve over time. Cross sectional studies also are unable to evaluate the correlation between BDNF level and duration and amount of alcohol usage. Several limitations in BDNF measurement in both plasma and serum are also additional concerns regarding the limitation of this study. Hence, caution should be exercised when interpreting our results.

## Conclusion

To conclude, this meta-analysis has demonstrated a significant correlation between alcohol consumption and decreased BDNF blood levels, with serum BDNF levels showing a stronger association than plasma BDNF levels. The study also suggests that BDNF levels may increase with longer withdrawal times. These findings shed light on the potential role of BDNF in the pathophysiology of alcohol dependence and can aid future research in this area. However, limitations especially due to the accuracy of measuring BDNF levels and the impact of other factors on BDNF levels guarantee the exercise of caution when interpreting our results. Further research with larger sample sizes especially longitudinal studies is needed to provide more robust evidence about cause-and-effect relationship between alcohol and BDNF. Investigation regarding genetics links of alcohol use and BDNF expression may also be elucidative regarding mechanisms involved in alcohol dependance. Finally, as the available data are limited and the findings uncertain, we encourage further investigations in order to provide more robust evidence.

### Supplementary Information

Supplementary Information.

## Data Availability

All data generated or analyzed during this study are included in this published article and its supplementary information files.
